# The Influence of a Wearable-Based Reward Program on Health Care Costs: Retrospective, Propensity Score–Matched Cohort Study

**DOI:** 10.2196/45064

**Published:** 2023-03-14

**Authors:** Amanda Zaleski, Brittany Sigler, Alan Leggitt, Shruti Choudhary, Ryan Berns, Kyu Rhee, Heidi Schwarzwald

**Affiliations:** 1 Clinical Evidence Development Aetna Medical Affairs CVS Health Hartford, CT United States; 2 Aetna Digital Product Development CVS Health Wellesley, MA United States; 3 Aetna Analytics & Behavior Change CVS Health New York, NY United States; 4 Aetna Medical Affairs CVS Health Hartford, CT United States

**Keywords:** digital health intervention, mobile app, wellness, physical activity, wearable, cost-effectiveness, mobile health app, health plan, medical cost, health care cost

## Abstract

**Background:**

Mobile health (mHealth) technology holds great promise as an easily accessible and effective solution to improve population health at scale. Despite the abundance of mHealth offerings, only a minority are grounded in evidence-based practice, whereas even fewer have line of sight into population-level health care spending, limiting the clinical utility of such tools.

**Objective:**

This study aimed to explore the influence of a health plan–sponsored, wearable-based, and reward-driven digital health intervention (DHI) on health care spending over 1 year. The DHI was delivered through a smartphone-based mHealth app available only to members of a large commercial health plan and leveraged a combination of behavioral economics, user-generated sensor data from the connected wearable device, and claims history to create personalized, evidence-based recommendations for each user.

**Methods:**

This study deployed a propensity score–matched, 2-group, and pre-post observational design. Adults (≥18 years of age) enrolled in a large, national commercial health plan and self-enlisted in the DHI for ≥7 months were allocated to the intervention group (n=56,816). Members who were eligible for the DHI but did not enlist were propensity score–matched to the comparison group (n=56,816). Average (and relative change from baseline) medical and pharmacy spending per user per month was computed for each member of the intervention and comparison groups during the pre- (ie, 12 months) and postenlistment (ie, 7-12 months) periods using claims data.

**Results:**

Baseline characteristics and medical spending were similar between groups (*P*=.89). On average, the total included sample population (N=113,632) consisted of young to middle-age (mean age 38.81 years), mostly White (n=55,562, 48.90%), male (n=46,731, 41.12%) and female (n=66,482, 58.51%) participants. Compared to a propensity score–matched cohort, DHI users demonstrated approximately US $10 per user per month lower average medical spending (*P*=.02) with a concomitant increase in preventive care activities and decrease in nonemergent emergency department admissions. These savings translated to approximately US $6.8 million in avoidable health care costs over the course of 1 year.

**Conclusions:**

This employer-sponsored, digital health engagement program has a high likelihood for return on investment within 1 year owing to clinically meaningful changes in health-seeking behaviors and downstream medical cost savings. Future research should aim to elucidate health behavior–related mechanisms in support of these findings and continue to explore novel strategies to ensure equitable access of DHIs to underserved populations that stand to benefit the most.

## Introduction

Chronic diseases are among the leading causes of death, disability, and excess medical spending in the United States and around the world. Approximately 60% of US adults have at least one chronic disease and contribute to 90% of the nation’s US $4.1 trillion in health care expenditures per year [1]. Individual self-management is recognized as a critical component for the prevention and management of high-cost, highly prevalent conditions and diseases such as diabetes mellitus and hypertension [2-4].

Active participation in care activities such as self-monitoring, preventive screenings, and lifestyle modifications bolsters self-efficacy and reinforces the prioritization of health. In addition, self-management narrows the gap between structured, patient-provider interactions by promoting ongoing collaboration and support toward health care goals, which ultimately alleviates pressure on the health care system [5].

Person-centered programs that are individually tailored to individual preferences, health circumstances, and lived environment are critical for the success of these care models, although scalability has been recognized as a key challenge in practice [6].

Mobile health (mHealth) technology is often touted as an easily accessible and cost-effective solution to support population-level health behavior change interventions at scale [7]. Digital platforms, connective devices such as wearables, and mHealth apps allow for the continuous collection of real-world data under conditions of daily living. This information, combined with self-reported data, can be leveraged to generate insights for personalized, just-in-time interventions using evidence-based behavior change techniques [7-9]. Rewards or incentives can elicit incremental, beneficial changes in key behaviors such as physical activity [10].

Despite promising results, there are known gaps identified in the literature that limit widespread mHealth adoption. Intervention components and hard clinical outcomes are vastly underreported, and a majority of trials are short in duration, which calls into question the likelihood for sustained engagement, behavior change, and durability of key outcomes of interest [11,12]. Lastly, a majority of third-party vendors lack insight into individual-level health care spending, thus making the cost-effectiveness of such programs difficult to quantify [13].

This study explored the influence of a health plan–sponsored digital health intervention (DHI) on economic outcomes over 1 year. Briefly, the DHI is an incentive-based mobile app that delivers evidence-based, personalized fitness and health education based on demographic data, medical and pharmacy claims, and objectively measured wearable sensor data. It was hypothesized that users who engaged with the DHI would have lower medical spending compared to a propensity score–matched control cohort of health plan members who were eligible for, but did not use, the DHI.

## Methods

### Study Design

This study deployed a propensity score–matched, 2-group, and pre-post observational design. Retrospective demographic and administrative medical and pharmacy claims data were deidentified, aggregated, and analyzed to determine the impact of the DHI on total health care spending and health care use trends. Claims data included diagnoses, procedures, laboratory results, sites of care, provider information, service costs, and drug identifiers. Claims data also included aggregations of the above information in the forms of medical cases, episode treatment groups, chronic condition flags, and predictive risk scores.

Demographic information collected during health insurance enrollment included self-reported sex, age, race, ethnicity, plan benefit details, location, and census tract statistics. Note that pharmacy claims data were only available for participants with data-sharing agreements between their pharmacy benefits manager and the health plan.

### Ethics Approval

Sterling Institutional Review Board reviewed and approved the study (#9882) as an exempt study under 45 CFR 46.104(d)(4). In addition, a waiver of Health Insurance Portability and Accountability Act authorization for the use and disclosure of aggregated, deidentified member data was obtained. No compensation was provided.

### Participants

All study participants were >18 years of age and enrollees of commercial health plans whose benefit structure included access to the DHI. All eligible members for the DHI found the program through either direct mail and email outreach, digital channels (eg, paid search, paid social, and programmatic), websites (via search engine optimization), media, SMS text messages, employer (ie, plan sponsor) internal promotion to their eligible employees, other channels based on existing preferences (eg, open enrollment materials, “next best actions” on health benefit web pages and apps, call-center scripting, and tags on medication packaging), or word of mouth. All DHI users voluntarily self-enlisted in the program and agreed to the terms and conditions upon logging into the app for the first time. Approximately 8 million enrollees were offered the DHI, of which 56,816 signed up during the program enlistment period.

Inclusion criteria included (1) continuous health plan eligibility throughout the study evaluation period (eg, from May 2019 to October 2020), (2) having administrative claims data for at least 1 year preceding the DHI enlistment (ie, “pre-enlistment”), and (3) having administrative claims data for ≥7 to 12 months after enlistment (ie, “postenlistment”). Potential participants were excluded if they did not meet the inclusion criteria or were <18 years of age.

The intervention group included DHI users who self-enlisted in the program and had at least one engagement touch point. The comparison group included commercial health plan members who were eligible for the DHI during the same study evaluation time period but did not enlist. Of note, participants were not excluded if they participated in other plan-sponsored programs with potential overlap, but this information was coded to ensure that the propensity score–matched groups were as equal as possible.

### Intervention Description

The DHI is a wearable- and smartphone-based mHealth app available only to members of a large commercial health plan offered since May 2019. The DHI leverages a combination of behavioral economics, user-generated sensor data from a connected wearable device, and claims history to create personalized, evidence-based recommendations for each user. User-generated data—retrieved from the wearable, mobile phone, and self-report—include heart rate, physical activity (eg, step counts, flights climbed, and estimated calories burned), estimated sleep quality, self-reported health-related measures (eg, nutrition, mindfulness activities, exercise type, height, weight, survey responses, and blood pressure), and health-related data collected from third-party apps with data-sharing permissions. Personalized daily and weekly messages are delivered through the smartphone app using a combination of lay education articles, short briefs, and video-based messaging.

The primary aim of the DHI is to engage users in their own health. Through this program, rewards are used as incentives, which can be redeemed as payments toward a connected, program-compatible wearable or gift cards. Users earn rewards by achieving weekly movement goals; completing objectively verifiable (ie, adjudicated) physical activity challenges; reviewing educational content around the 4 lifestyle behavior pillars of physical activity, nutrition, mindfulness, and sleep in the app; or completing recommended clinical actions based on the user’s demographic and health history (termed within the app as “Healthy Actions”). Healthy Actions are grounded in well-established, evidence-based guidelines for population health (eg, United States Preventive Services Task Force [14], Centers for Disease Control and Prevention [15], American Heart Association [3,16], American College of Sports Medicine [17], etc). Healthy Actions focus on primary and secondary prevention, including informing users where they can receive an influenza vaccination or how they can participate in relevant screenings or surveys. Each use case includes educational content that reinforces or references publicly available health education intended for lay audiences and is approved by an internal panel of medical directors. The average completion rate of Healthy Actions for all DHI users is 90%, with an average user satisfaction rate of 80%.

The DHI draws on several well-established health behavior theoretical constructs to inform program intervention components [7,12,18], including social cognitive theory and the health belief model [17]. Central to social cognitive theory is the concept of self-efficacy (ie, belief that one can successfully complete a task). Leveraging behavior change strategies such as goal setting, physiological feedback, reinforcement, and cointerventions (ie, rewards and incentives) foster positive outcome expectations that allow users to “link” their reward to their behavior, which reinforces self-efficacy and promotes sustained behavior change [17]. The health belief model posits that adults are most likely to engage in health-related behavior change if one believes they are at risk and that the health-related behavior change will effectively impact their risk [12,17]. As such, the DHI serves as a trusted guide through the provision of evidence-based, credibly sourced health education; strategies to overcome likely barriers; and personalized, relevant, and timely information to facilitate informed health care decisions.

### Statistical Analysis

A retrospective, propensity score–matched cohort approach was used to estimate the causal effects of enlistment in the DHI. Propensity score–matching is the best-in-class approach to reduce potential selection bias and confounding variables inherent in studies that are unable to deploy a randomized controlled trial design (such as the case in most plan-sponsored DHIs) [19]. An ensemble model was trained to predict the likelihood of enlistment in the DHI within the eligible population. Features used to predict enlistment included demographic features (eg, age and sex), health plan details (eg, fully insured vs self-insured and types of coverage), communication receptivity (eg, member permission to email, text, and provide in-app notifications), medical and pharmacy use (eg, diagnosis codes, procedure codes, inpatient visits, and emergency department [ED] visits), and chronic conditions (eg, hypertension and diabetes). For the intervention group, a 1-year window prior to the DHI enlistment date was selected as the feature window. For the comparison cohort group, an iterative process was applied.

Features were created for each month of engagement, matches were assigned, and then the unmatched comparisons were designated for matching enrolled users for the next month. To further balance the intervention and comparison groups, matches were assigned within specific segments of the cohort based on self-reported sex, pharmacy data availability, and health plan type (eg, fully insured vs self-insured). Members of the comparison group were matched to the intervention group using an iterative nearest neighbor approach, resulting in 1:1 matching. After matching, balance between groups was confirmed by checking for differences between groups across several features including demographic features, location (eg, urban, suburban, or rural zip code), overlap with other health plan programs (eg, care management and member advocacy programs), member risk (eg, predicted use score and number of chronic diseases), acute medical events during the pre-enlistment period (eg, number of inpatient and ED visits), and baseline absolute medical and pharmacy use (proprietary; data not shown). No significant differences were observed across these factors.

Claims data were used to compute average medical and pharmacy spending per user per month (PUPM) for each member of the intervention and comparison groups during the pre- and postenlistment periods. Health care spending was excluded from the analysis if it was deemed nonimpactable or pregnancy related. Nonimpactable spending was assessed using a proprietary diagnosis-related group schema, which considers the diagnosis and procedure codes associated with a visit to an inpatient or nonacute facility. Total individual health care spending was capped at the 99th percentile for both the pre-enlistment and postenlistment periods. Difference in average monthly spending was calculated as postenlistment monthly spending minus the pre-enlistment monthly spending. Two-tailed paired *t* tests were used to test differences between the pre-enlistment and postenlistment periods within each population. Two-tailed unpaired *t* tests were used to explore differences between the intervention (ie, the DHI) and comparison groups. Statistical significance was defined as *P*<.05.

Cost category analysis was conducted by segmenting medical costs into the following subcategories: ED, specialist, mental health, inpatient admissions, radiology, primary care, ambulatory care, laboratory, home health, medical pharmacy, and other pharmacy. ED admissions were further classified as “emergent” or “nonemergent” according to the New York University ED visit algorithm—the most widely used tool for retrospectively assessing the probability that ED visits are urgent, preventable, or optimally treated in an ED based on administrative claims data [20].

## Results

Table 1 details baseline user characteristics of the intervention and comparison groups before and after propensity score matching. Prior to matching (and compared to matched cohorts), DHI users were more likely to be younger in age, female, have fewer chronic conditions, and exhibit fewer ED and inpatient visits. After propensity score matching, the comparison group exhibited similar baseline characteristics to the DHI-enrolled population. On average, the total included sample population (N=113,632) consisted of young to middle-age (mean age 38.81 years), mostly White (n=55,562, 48.90%), male (n=46,731, 41.12%) and female (n=66,482, 58.51%) participants, of which 35.66% (n=40,519) had at least one chronic condition.

**Figure 1:**
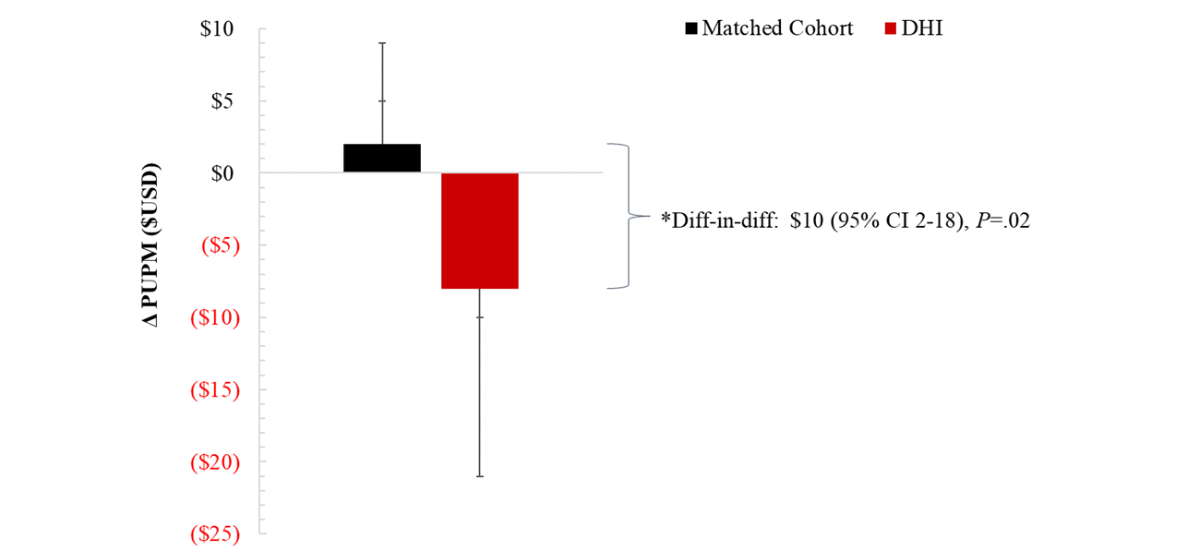
Average change in health care spending PUPM between DHI users and matched comparison cohorts before and after DHI enlistment. Diff-in-diff: difference-in-differences; DHI: digital health intervention; PUPM: per user per month; USD: US dollars.

Average and relative change in health care spending for medical and pharmacy claims were calculated for the pre- and postenlistment periods. At baseline, both groups exhibited similar spending (*P*=.89). In the postenlistment period, average spending was unchanged for the comparison group (mean change US $2 PUPM, 95% CI –US $3 to US $7; *P*=.44), whereas spending decreased (–US $8 PUPM, 95% CI –US $13 to –US $2; *P*=.04) for DHI users with a difference-in-difference of US $10 PUPM (95% CI US $2 to US $18; *P*=.02; Figure 1).

To better characterize the overall reduction in health care costs, an analysis of cost breakdown by categories of medical spending was conducted over the same pre- and postenlistment periods (Table 2). Notably, there was an observed reduction in ED-, specialist-, and mental health–related medical spending. Of note, there was an observed trend of increased spending related to primary care; however, this did not achieve statistical significance (*P*=.09).

**Table 1:** Baseline demographic and use data in the unmatched and propensity score–matched cohorts.

Characteristic				Unmatched cohort			Propensity score–matched cohort	
			Total eligible (n=8,504,571)		DHI^a^ (n=56,816)	Matched comparison (n=56,816)		DHI (n=56,816)
**Demographic**								
	Age (years), mean		42.37		37.83	37.21		37.83
	**Sex, n (%)**							
		Male	4,073,158 (47.89)		23,446 (41.27)	23,443 (41.26)		23,446 (41.27)
		Female	4,429,950 (52.09)		33,368 (58.73)	33,368 (58.73)		33,368 (58.73)
		Nonbinary	463 (0.01)		2 (0)	5 (0.01)		2 (0)
	**Location, n (%)**							
		Urban	3,334,579 (39.21)		20,803 (36.61)	21,466 (37.78)		20,803 (36.61)
		Suburban	2,034,110 (23.92)		15,657 (27.56)	16,004 (28.17)		15,657 (27.56)
		Rural	3,013,633 (35.44)		20,240 (35.62)	19,228 (33.84)		20,240 (35.62)
	**Race/ethnicity, n (%)**							
		Alaska Native or American Indian	22,946 (0.27)		219 (0.39)	207 (0.36)		219 (0.39)
		Asian	365,816 (4.30)		4164 (7.33)	3979 (6.99)		4164 (7.33)
		Black or African American	383,331 (4.51)		3393 (5.97)	4104 (7.22)		3393 (5.97)
		Missing race data	3,333,979 (39.21)		9997 (17.60)	10,955 (19.28)		9997 (17.60)
		Multiple races	391,787 (4.61)		3077 (5.42)	3484 (6.13)		3077 (5.42)
		Native Hawaiian or Other Pacific Islander	10,232 (0.12)		106 (0.19)	101 (0.18)		106 (0.19)
		Other	123,714 (1.45)		865 (1.52)	1217 (2.14)		865 (1.52)
		Unanswered	902,934 (10.62)		5548 (9.76)	5512 (9.70)		5548 (9.76)
		Unknown	296,307 (3.49)		597 (1.05)	612 (1.08)		597 (1.05)
		White	2,672,525 (31.43)		28,850 (50.78)	26,651 (46.91)		28,850 (50.78)
**Chronic conditions, n (%)**								
	1		3,512,921 (41.31)		20,450 (35.99)	20,213 (35.58)		20,450 (35.99)
	≥2		2,182,191 (25.66)		10,375 (18.26)	10,048 (17.68)		10,375 (18.26)
**Acute events (per 1000 members), n**								
	Inpatient visits		251		161	159		161
	ED^b^ visits		198		181	181		181
**Other plan benefits, n (%)**								
	Care management		865,664 (10.18)		9897 (17.42)	9971 (17.55)		9897 (17.42)
	Member advocacy programs		427,730 (5.03)		7363 (12.96)	7312 (12.87)		7363 (12.96)

^a^DHI: digital health intervention.

^b^ED: emergency department.

**Table 2:** Difference in costs per medical cost category in digital health intervention users (from matched cohorts).

Medical cost category	PUPM^a^ savings (US $), mean change (95% CI)	*P* value
ED^b^	5.7 (3 to 8)	.001
Emergent ED	2.6 (–5 to 8)	.62
Nonemergent ED	3.2 (–1 to 4.6)	.07
Mental health	1.7 (0.3 to 3)	.01
Specialist	2.7 (0.1 to 5)	.04
Primary care	–0.4 (–0.8 to 0.1)	.09
Laboratory	–0.0 (–1 to 1)	.09
Radiology	1.0 (–0.4 to 2)	.16
Inpatient	1.0 (–0.9 to 10)	.89
Ambulatory	–0.3 (–4 to 3)	.88
Home health	0.2 (–0.2 to 0.7)	.38
Medical Rx^c^	0.3 (–2 to 2)	.78
Total Rx	–1.4 (–4 to1)	.37
Overall PUPM savings	10.0 (2 to 18)	.02

^a^PUPM: per user per month.

^b^ED: emergency department.

^c^Rx: prescription.

## Discussion

### Principal Findings

This study sought to explore the influence of an incentive-based mHealth intervention with wearable integration on health care costs over 1 year. As hypothesized, medical spending was approximately US $10 PUPM lower in DHI users compared to a propensity score–matched cohort. Put into context, these savings translate to approximately US $6.8 million in avoidable health care costs for the included study sample of 56,816 users over the course of 1 year. In addition, DHI users exhibited favorable changes in health-related behaviors, including decreased use of high-cost, preventable services (ie, nonemergent ED admissions) and decreased spending related to mental health and specialty visits. Of note, there was a trend toward marginally increased spending related to primary care (*P*=.09), but these results were not statistically significant.

### Interpretation of Principal Findings

Mechanisms to explain our findings are likely multifactorial. At its core, the multicomponent mHealth intervention combines several self-management techniques at varying intensities, including knowledge acquisition, enhancing decision-making skills (ie, optimal sites of care for nonemergencies), stimulation of independent health monitoring, medication adherence, and lifestyle behavior change [21]. Given the holistic nature of these microinterventions, members engaged with the DHI are likely to experience additive benefits above and beyond the intended outcomes owing to a single intervention alone. In addition, a majority (ie, 60%) of US adults have at least one chronic condition, and 42% have multiple chronic conditions [22]. Beneficial outcomes are likely “magnified” in members with multiple conditions amenable to one (or more) shared intervention.

The observed reduction in nonemergent ED admissions is directly aligned with DHI-related campaigns to inform members of appropriate alternative site-of-care categories available for nonemergent health problems (Multimedia Appendix 1). Similarly, this DHI continuously reinforces the member-provider relationship through regular prompts and reminders to close preventive gaps in care. This messaging aligns with findings that signal slightly increased medical spending for primary care encounters. One unexpected outcome was that DHI users spent on average approximately US $1.7 PUPM less on mental health services than non-DHI users. It is possible that key health behaviors (ie, physical activity and medication adherence) translated to clinically meaningful changes in mental health and well-being; however, this is speculative.

Although this study was not designed to explore the impact of relative changes in physical activity levels on health care costs, preliminary post hoc subgroup analysis revealed a signal to this effect. Among participants who increased their average daily caloric expenditure by at least 50 kilocalories over the course of DHI use, there was an observed reduction in medical costs on the order of US $39 PUPM compared to their propensity score–matched comparison cohorts (*P*<.001). Similarly, post hoc analyses revealed a correlation between program engagement and varying degrees of medical cost savings of greater magnitude. These findings are promising and warrant additional exploration into what levers (eg, engagement, education, physical activity, and preventive care reminders) are the most likely to influence health care spending and for whom.

### Limitations

There are few noteworthy limitations to this study. This study infers cost savings to be driven by changes in health-seeking behavior. Although these findings were statistically significant, this study was not designed to explore a definitive causal relationship. Randomized controlled trials are the gold-standard approach to measure the causal effectiveness of an intervention but are not feasible to conduct for health plan–sponsored benefits [19]. Retrospective observational comparator studies are a statistically valid approach to test the real-world effectiveness of low-risk DHIs in large, heterogenous populations [23]. Although subject to selection bias and confounding variables, propensity score approaches allow for the comparability of preintervention covariates and control for any potential confounding bias in reported outcomes [19].

The time horizon selected for analysis was prior to the COVID-19 pandemic and does not reflect member behavior in a postpandemic era. Postpandemic data were intentionally excluded from analyses as COVID-19–related health behaviors are continuously evolving. However, we hypothesize that the nature of results would remain the same—and likely be even *greater* in magnitude. Indeed, recent surveys indicate health to be of increasing importance to US adults [24], thus engagement and outcomes owing to DHIs, such as the one presented here, are likely superior in a pre- versus postpandemic era. Finally, of those who reported race (82,168/113,632, 72.31%), a majority (55,562/82,168, 67.62%) of the studied population was White. Although there was some representation from Black or African American, Asian, Alaska Native or American Indian, Native Hawaiian or Other Pacific Islander, Other, and multiple races populations, these numbers do not reflect the diversity of the US population and limit the generalizability of these results. Current efforts are underway to improve the collection of race and ethnicity data—a critical next step to advance health equity.

Despite the noted limitations, this study possesses several strengths. This study is a rigorously designed retrospective, propensity score–matched cohort study to explore health care spending in a large, geographically diverse sample size of 113,632 individuals. A major strength of this study is the integration of additional member data typically unaccounted for in such approaches (eg, plan benefit structure and overlapping programs). Indeed, *t* tests conducted on final propensity score–matched groups indicated that efforts to minimize confounding bias were successful. Additionally, there are limited studies that examine the cost-effectiveness of behavior change and physical activity–based apps [25,26].

This study adds to the body of literature with its evaluation of the impact of combining mHealth-connected wearables with employer- or plan-sponsored incentives. Access to claims data enrich the results that can be elicited, including the quantification of real-world cost savings without having to be anchored on simulated models or estimates. This DHI is grounded in evidence-based practice and informed by health behavior theoretical constructs and behavior change strategies. Finally, the study cohort is a representative population for whom such an intervention would be used by (ie, members of a large, commercial health plan). As the use of wearable devices and their functionality continues to evolve, this study highlights early indicators of success via the combination of mHealth-connected wearables with employer-sponsored incentives and payor data.

### Future Research

Future research to elucidate mechanistic underpinnings for decreased medical spending exhibited by users in this, or other similar, DHIs would be of great public health interest. Relatedly, identifying the impact of DHIs on patient-centered outcomes and other health-related benefits will allow researchers to fully appraise the return on investment for overall health. In addition, the development of predictive models can potentially enhance early identification of risks for common cardiovascular and behavioral health conditions. Although DHIs are not intended to diagnose, treat, or substitute for provider advice, earlier detection of potential disease onset or deterioration can be used to inform condition-specific educational outreach tailored to end users. Additionally, researchers are well poised to broaden the reach of DHIs by expanding access to multiple platforms, offering “bring your own device” programs, addressing key social determinants of health, and ensuring equitable access for underserved populations.

### Conclusions

The study demonstrates that this incentive-based DHI translates to approximately US $120 per user, per year in medical cost savings compared to a propensity score–matched comparison cohort. These findings indicate that this, and other similar, DHIs hold great promise as an effective strategy to improve population health at large.

## Appendix

Multimedia Appendix 1 Digital health intervention screenshots showing 2 uses cases to support informed decision-making for emergent versus nonurgent sites of care and scheduling an annual wellness exam.

## Abbreviations

DHI: digital health intervention
ED: emergency department
mHealth: mobile health
PUPM: per user per month

## Data Availability

The data sets generated during and/or analyzed during the current study are not publicly available due to commercial data use agreements.
