# Supplementary figures and images for: The Influence of a Wearable-Based Reward Program on Health Care Costs: Retrospective, Propensity Score–Matched Cohort Study
Source: J Med Internet Res. 2023 Mar 14;25:e45064. doi: 10.2196/45064 (PMC10131601; doi:10.2196/45064)

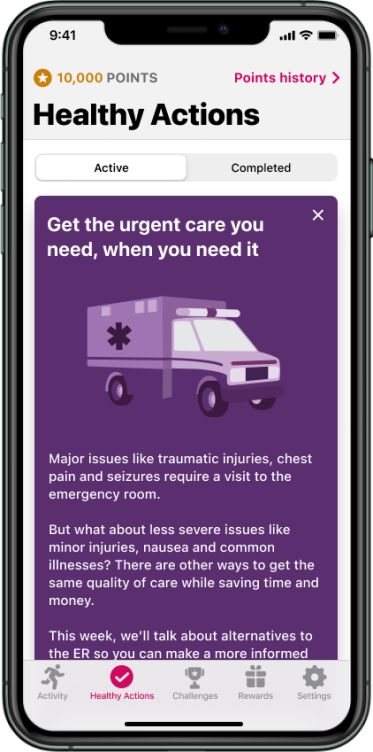

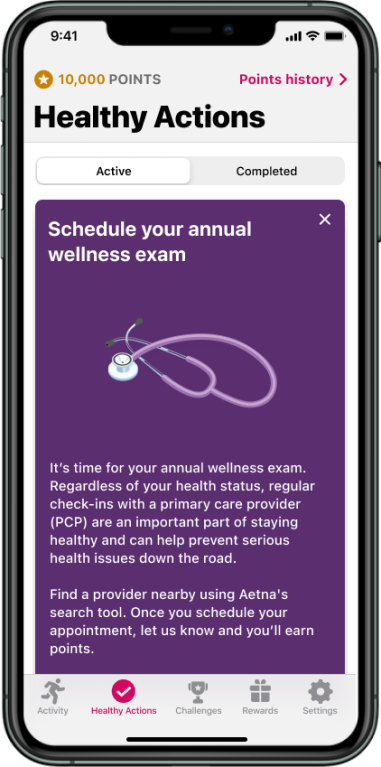

Supplement: Multimedia Appendix 1 [file jmir_v25i1e45064_app1.docx]
